# Pyroptosis relates to tumor microenvironment remodeling and prognosis: A pan-cancer perspective

**DOI:** 10.3389/fimmu.2022.1062225

**Published:** 2022-12-20

**Authors:** Muhammad Khan, Meiling Ai, Kunpeng Du, Jingjing Song, Baiyao Wang, Jie Lin, Anbang Ren, Chengcong Chen, Zhong Huang, Wenze Qiu, Jiangyu Zhang, Yunhong Tian, Yawei Yuan

**Affiliations:** ^1^ Department of Radiation Oncology, Affiliated Cancer Hospital and Institute of Guangzhou Medical University, Guangzhou, China; ^2^ State Key Laboratory of Respiratory Diseases, Guangzhou Institute of Respiratory Disease, Guangzhou Medical University, Guangzhou, China; ^3^ Department of Pathology, Affiliated Cancer Hospital and Institute of Guangzhou Medical University, Guangzhou, China

**Keywords:** pyroptosis, pancancer, genomic variation, DNA methylation, prognosis, immune cell infiltration

## Abstract

**Background and aim:**

Pyroptosis is an inflammatory form of programmed cell death implicated in inflammation and disease. Moreover, inducing pyroptosis has been appreciated as anti-cancer therapy for its ability to unleash anti-cancer immune responses.

**Methods:**

Utilizing the data available in The Cancer Genome Atlas (TCGA), pyroptosis-related genes’ (PRGs) expression, genomic aberrations, and clinical significance were systematically analyzed in pan-cancer. A GSVA score was obtained to rate pyroptosis level and divide the cancers into pyroptosis-low and pyroptosis-high groups. Immunohistochemistry (IHC) was used to evaluate the differential expression of major PRGs (GSDMC, GSDMD, GSDME, NLRP3, NLRC4, IL1B) in selected tumor types (COAD, HNSC, KIRC, LIHC, LUAD, LUSC). Selection of tumors for immunohistochemistry (IHC) was based on their expression pattern in TCGA cancers, clinical relevance, tumor epidemiology, and sample availability.

**Results:**

Differential expression of PRGs was evident in various cancers and associated with prognosis which was driven by genomic variations and epigenetic abnormalities, such as single nucleotide variations (SNVs), copy number variation (CNV) and DNA methylation level. For example, methylation of PRGs in lower grade glioma (LGG), uveal melanoma (UVM) and kidney renal clear cell carcinoma (KIRC) were predictive of improved survival as upregulation of PRGs was risky in these cancers. Pyroptosis level significantly differentiated tumor from normal samples in 15 types of cancers, exhibited a progressive trend with cancer stage, observed variation among cancer subtypes, and showed a significant association with cancer prognosis. Higher pyroptosis level was associated with worst prognosis in majority of the cancers in terms of OS (KIRC, LGG, and UVM), PFS (GBM, KIRC, LGG, PRAD, THCA, and THYM) and DSS (KIRC and LGG) as estimated by Kaplan-Meier survival curves. Moreover, Pyroptosis level was strongly indicative of a hot tumor immune microenvironment with high presence of CD8+ T cell and other T cell subtypes. Several oncogenic pathways, such as P53 pathway, DNA repair, KRAS signaling, epithelial-mesenchymal transition (EMT), IL6 JAK STAT3 signaling, IL2 STAT5 signaling, PI3K AKT MTOR signaling and angiogenesis, were enriched in pyroptosis-hi subgroups across cancers.

**Conclusions:**

Genetic alterations in PRGs greatly influence the pyroptosis level and cancer prognosis. A relatively hot tumor immune microenvironment was associated with pyroptosis irrespective of the cancer prognosis. Overall, our study reveals the critical role of pyroptosis in cancer and highlights pyroptosis-based therapeutic vulnerabilities.

## Introduction

Pyroptosis (pyro- Greek for fire, and ptosis, falling) is an inflammatory form of programmed cell death prompted by microbial infective agents and endogenous danger signals (1, 2). Pyroptosis is characterized by the activation of inflammasomes, development of pores in the plasma membrane by gasdermins (GSDM protein superfamily members), and maturation and release of pro-inflammatory cytokines interleukin-1β and IL-18 (1, 3). Key players of pyroptosis include: nod-like receptors (NLRs) associated with inflammasomes formation; and gasdermins which mediate plasma membrane pore formation. The latter being considered as the primary executioner of pyroptosis (1–3). Inflammasome-associated NLRs include NLRP1, NLRP3, NLRC4, AIM2 and PYRIN (4, 5). Gasdermins are widely expressed and comprised of GSDMA, GSDMB, GSDMC, GSDMD, and GSDME with GSDMD and GSDME being the most studied (6–8). Gasdermin activation may or may not involve inflammasome formation based on the receiving signal. In canonical pathway, PAMPs (Pathogen‐associated molecular pattern molecules), and DAMPs (damage‐associated molecular pattern molecules) stimulation cause NLRs, pro-caspase-1 and apoptosis-associated speck-like protein (ASC) assembly resulting in caspase-1 activation. Caspase-1 cleaves GSDMD, pro-IL-1β and IL-18 (9). In non-canonical pathway, caspase-4/5/11 are directly activated by invading agents/endogenous factors resulting in GSDMD-mediated pore formation and cell death (10). Granzymes, granzyme A and B (GZMA, GZMB), are also indicated in direct cleavage of gasdermin proteins (11, 12). Gasdermin cleavage by caspases/granzymes results in dissociation of pore-forming domain of the N-terminal and repressor domain of the C-terminal followed by oligomerization of the N-terminal pore-forming domain and pore formation in the plasma membrane (6, 13).

Pyroptosis mechanistic revelations have enhanced our biological understanding of this process in inflammation and disease. It has closely been linked to several diseases of diverse nature, such as infectious, neurological, cardiovascular, autoimmune diseases, and cancer (14–17). In cancer, pyroptosis has shown suppression as well as promotion of tumorigenesis (18). This behavior has been extended to both of its key actors: inflammasomes; and gasdermins. For example, NLRP1 was implicated in the promotion of melanoma tumorigenesis through caspase-1 mediated inflammasome activation but also inhibiting caspase-2/9 related mitochondrial apoptosis (19). Similarly, in the absence of NLRP3, numbers of activated NK cells were increased with more IFN-γ secretion and killing of tumor cells to reduce B16F10 lung metastasis (20). On the other hand, *Nlrp1b^−/−^*, *Nlrp3^−/−^*, *Nlrc4^−/−^*, *Aim2^−/−^*, and *pyrin^−/−^* mice were more susceptible to increase in inflammation, morbidity, and tumorigenesis as compared to wild-type mice (21–25). Likewise, gasdermins have also exhibited complex behavior; as tumor suppressor genes as well as oncogenes (7). Gasdermin B, C and D have shown contrast behavior in cancers (26–33). *GSDMB* amplification is observed in hepatic, cervical, colon and HER2+ breast cancer suggestive of oncogenic tendency (26, 27). Nonetheless, its suppression is reported in esophageal and gastric tumors (12, 28). Moreover, patients with bladder cancer and melanoma observed better prognosis with high expression of *GSDMB* (12). *GSDMC* also suppresses gastric cell proliferation but upregulation is observed in certain cancers such as melanoma, colorectal and lung adenocarcinoma which is associated with tumor growth and metastasis (28–31). Likewise, suppression of gastric cell proliferation is also observed with GSDMD but reduced expression was associated with mitigation of tumor proliferation and favorable prognosis in non-small cell lung cancer (28, 32, 33). GSDME is rather considered as an immunosuppressant (34–36). Hence, gasdermins expression and function is cancer-specific and context based. More importantly, inducing pyroptosis has been evaluated for its therapeutic implications in cancer treatment (37). For example, pyroptosis of less than 15% of tumor cells was enough to eradicate 4T1 mammary tumor graft *via* augmentation of immune responses (38).

Given the complex dynamics and therapeutic importance of pyroptosis in cancer, we have performed a comprehensive genomic and functional analysis of pyroptosis-related genes (PRGs) in pan-cancer. Impact on clinical features, immune microenvironment, drug vulnerabilities and prognosis were also computationally established. The results indicate a critical role of PRGs in pan-cancer and provide a platform for further cancer-specific exploration of pyroptosis.

## Methods

### Data and sources

The Cancer Genome Atlas (TCGA) (https://portal.gdc.cancer.gov/) database was explored to obtain transcriptome profiling (n=10, 476) and associated clinical data (n=11, 315) for 33 types of cancers including adrenocortical carcinoma (ACC), bladder urothelial carcinoma (BLCA), breast invasive carcinoma (BRCA), cervical and endocervical cancers (CESC), cholangiocarcinoma (CHOL), colon adenocarcinoma (COAD), Lymphoid neoplasm diffuse large B-cell lymphoma (DLBC), esophageal carcinoma (ESCA), glioblastoma multiforme (GBM), head and neck squamous cell carcinoma (HNSC), kidney chromophobe (KICH), kidney renal clear cell carcinoma (KIRC), kidney renal papillary cell carcinoma (KIRP), acute myeloid leukemia (LAML), Lower grade glioma (LGG), liver hepatocellular carcinoma (LIHC), lung adenocarcinoma (LUAD), lung squamous cell carcinoma (LUSC), pancreatic adenocarcinoma (PAAD), mesothelioma (MESO), ovarian serous cystadenocarcinoma (OV), pancreatic adenocarcinoma (PAAD), pheochromocytoma and paraganglioma (PCPG), prostate adenocarcinoma (PRAD), rectum adenocarcinoma (READ), sarcoma (SARC), skin cutaneous melanoma (SKCM), stomach adenocarcinoma (STAD), testicular germ cell tumors (TGCT), thyroid carcinoma (THCA), thymoma (THYM), uterine corpus endometrial carcinoma (UCEC), uterine carcinosarcoma (UCS), and uveal melanoma (UVM). Other data collected included single nucleotide variation (SNV) data (n=10, 234), copy number variation (CNV) data (n=11, 495), and DNA methylation data (n=10, 129). Gene expression in the various normal tissues was analyzed using The Genotype-Tissue Expression (GTEx) dataset (V8.0) (https://commonfund.nih.gov/GTEx/) which comprises 15, 253 samples from 54 tissues of 838 healthy individuals. Genemania (https://genemania.org/) database was utilized to obtain the gene interaction network. Protein-protein interaction (PPI) network and co-expression for PRGs were constructed with the Search Tool for the Retrieval of Interacting Genes (STRING) database, version 11.5 (https://string-db.org/). Half-maximal inhibitory concentration (IC50) data (z-scored) of FDA-approved drugs were downloaded from the CellMiner database (https://discover.nci.nih.gov/cellminer).

### Identification of pyroptosis-related genes

We identified 31 pyroptosis-related genes summarized in the previous studies (1, 8, 38). These genes included: the Gasdermin (GSDM) family genes (*GSDMA, GSDMB, GSDMC, GSDMD, GSDME* (also known as *DFNA5* [Deafness, autosomal dominant, 5]), *PJVK* (Pejvakin; also known as *DFNB59* [Deafness, autosomal recessive, 59]); the NLRs (node-like receptors) family (*NLRP1* [Pyrin Containing 1], *NLRP3, NLRP9, NLRC4* [CARD Containing 4], and *AIM2* [Absent in melanoma 2]); the Caspase (CASP) family members (*CASP1, CASP4, CASP5, CASP6, CASP8*, and *CASP11* (also known as *SCAF11* [SR-Related CTD Associated Factor 11]); the pro-inflammatory cytokines (*IL-1B* [Interleukin 1 beta] and *IL-18* [Interleukin 18]); and other relevant pathway regulators including *ZBP1* (Z-DNA-binding protein 1), *PYCARD* (PYD And CARD Domain Containing; also known as *ASC* [Apoptosis-associated speck-like protein containing a CARD]), *GZMB* (Granzyme B), *GZMA* (Granzyme B), *PRF1* (Perforin 1), *CPTP* (Ceramide-1-Phosphate Transfer Protein; also known as *GLTPD1* [Glycolipid Transfer Protein Domain-Containing Protein 1]), *DHX9* (DExH-Box Helicase 9), *NAIP* (NLR Family Apoptosis Inhibitory Protein), *DDX3X* (DEAD-Box Helicase 3 X-Linked), *APIP* (APAF1 Interacting Protein), *CTSG* (Cathepsin G) and *GPX4* (Glutathione peroxidase 4).

### Gene expression in normal tissues

Tissue-specific gene expression of pyroptosis-related was carried out using GTEx Portal. Gene expression was normalized using the Transcripts Per Million (TPM).

### Differential gene expression

Differential expression analysis was carried out to determine the PRGs differentially expressed between cancer and normal tissues. Gene expression (mRNA Seq) data for 33 cancer types involving 10, 469 samples was obtained from TCGA database in March 2022 (**Table 1**). Analyses were restricted to 18 cancer types including BLCA, BRCA, CHOL, COAD, ESCA, GBM, HNSC, KICH, KIRC, KIRP, LIHC, LUAD, LUSC, PRAD, READ, STAD, THCA, and UCEC, which possessed paired tumor and normal samples. Fold changes were obtained by dividing means (tumor/normal), and the p-value was calculated using Wilcoxon rank test. DEGs were defined as genes with a logFC=0.5 and p value less than 0.05.

**Table 1:** The number of samples and abbreviations for the 33 types of tumors explored in this study.

Tumor	Abbreviation	Number of samples
Adrenocortical carcinoma	ACC	79
Bladder urothelial carcinoma	BLCA	427
Breast invasive carcinoma	BRCA	1218
Cervical squamous cell carcinoma and endocervical adenocarcinoma	CESC	310
Cholangiocarcinoma	CHOL	45
Colon adenocarcinoma	COAD	329
Lymphoid neoplasm diffuse large B-cell lymphoma	DLBC	48
Esophageal carcinoma	ESCA	196
Glioblastoma multiforme	GBM	173
Head and neck squamous cell carcinoma	HNSC	566
Kidney chromophobe	KICH	91
Kidney renal clear cell carcinoma	KIRC	606
Kidney renal papillary cell carcinoma	KIRP	323
Acute myeloid leukemia	LAML	173
Lower grade glioma	LGG	534
Liver hepatocellular carcinoma	LIHC	424
Lung adenocarcinoma	LUAD	576
Lung squamous cell carcinoma	LUSC	553
Mesothelioma	MESO	87
Ovarian serous cystadenocarcinoma	OV	309
Pancreatic adenocarcinoma	PAAD	183
Pheochromocytoma and paraganglioma	PCPG	187
Prostate adenocarcinoma	PRAD	550
Rectum adenocarcinoma	READ	105
Sarcoma	SARC	265
Skin cutaneous melanoma	SKCM	474
Stomach adenocarcinoma	STAD	450
Testicular germ cell tumors	TGCT	156
Thyroid carcinoma	THCA	572
Thymoma	THYM	122
Uterine corpus endometrial carcinoma	UCEC	201
Uterine carcinosarcoma	UCS	57
Uveal melanoma	UVM	80

### Gene set variation analysis

Gene set variation analysis (GSVA) scores individual samples for gene sets by estimating variation of gene set enrichment through the samples of an expression dataset in an unsupervised manner. “R” package GSVA was utilized to calculate GSVA-score which represented integrated-level of PRGs’ expression and was defined as the pyroptosis level to computationally dissect the tissue samples into high and low pyroptotic samples (39).

### Gene set enrichment analysis

Gene set enrichment analysis (GSEA) was performed to identify the cancer pathways associated with pyroptosis (40). Based on the pyroptosis level, the tumor samples were divided into low- (bottom 30%) and high-pyroptosis level groups (top 30%).

### Analysis of clinical aspects

Prognostic significance of pyroptosis level across 33 cancer types was investigated by grouping tumor samples into low and high pyroptotic groups *via* median value of pyroptosis score (GSVA score). R package “survival” was used to assess the prognostic potential of the pyroptosis level among cancers employing both Kaplan–Meier and cox proportional-hazards model analysis. Prognostic parameters included: overall survival (OS); progression-free survival (PFS); and disease-specific survival (DSS).

### Cancer stage

Cancer stage data for 21 cancer types (ACC, BLCA, BRCA, CHOL, COAD, ESCA, HNSC, KICH, KIRC, KIRP, LIHC, LUAD, LUSC, MESO, PAAD, READ, SKCM, STAD, TGCT, THCA, UVM) was collected for cancer stage analysis. Two types of cancer staging data, such as pathologic and clinical which classified samples into Stage I, II, III, and IV, were evaluated. Clinical relevance of stage and GSVA score was evaluated with ANOVA test. A trend analysis was utilized to get the trend of expression (GSVA score) as the disease progresses. Trend analysis was performed using Mann-Kendall Trend Test. The p value of the trend analysis is for reference only as the p value for Mann-Kendall analysis depends on the number of subjects which is only four (no of stages=4) in this case – too small to get a significant p value.

### Subtype analysis

Subtype changes of gene set expression were sought using the clinical data of 11 cancer types (HNSC, LUSC, COAD, STAD, LUAD, GBM, BRCA, UCEC, KIRC, READ, BLCA). Wilcoxon (n=2) and ANOVA (n>2) tests were used as per the number of subgroups in a subtype. If available, subtype indicated molecular subtypes, otherwise clustered subtypes.

### Genomic aberrations

#### Single nucleotide variation analysis

The mutation frequency and oncoplot of 31 PRGs in 33 types of cancers were constructed by the “maftools” R package. Mutation frequency was calculated using the formula: number of mutated samples/number of cancer samples. Gene set SNV was calculated which indicated the integrated SNV status of the input PRGs for each sample. Tumor samples were divided into two groups based on the presence of deleterious mutations (Mutant versus WT). Mutant group was defined when at least one gene in the input gene set was mutated in the sample. R package survival was used to fit survival time and survival status within groups. Cox proportional-hazards model and log-rank tests were performed to test the survival difference between WT and Mutant groups.

#### Copy number variation analysis

Copy number variation (CNV) data comprising 11 495 samples was downloaded from TCGA database. GISTICS2.0, which identify significantly altered regions of amplification or deletion across sets of patients, was used for processing the CNV data. GISTICS2.0 ascribe a certain GISTIC score (-2, -1, 1, 2) according to the type of variation. Spearman’s correlation was performed to obtain the correlation between CNV and gene mRNA expression. The gene set CNV was calculated which indicated the integrated CNV status of the input PRGs for each sample. The samples were divided into WT, Amp. and Dele. groups. A sample was classified as “Amplified” or “Deleted” when at least one PRG was constantly amplified or deleted in this sample. Samples with inconsistent gene status (Gene A is amplified while gene B is deleted) were excluded from analyses. R package survival was used to fit survival time and survival status within groups. Cox proportional-hazards model and log-rank tests were performed to test the survival difference between the groups.

#### Methylation analysis

Methylation status (hypo- or hypermethylated) of each PRG for each sample (tumor/normal) was determined by Wilcoxon signed rank test with a p value less than 0.05. The R package “IlluminaHumanMethylation-450kanno.ilmn12.hg19” was incorporated to annotate the methylation probe for the promoter of each gene. Correlation between PRGs expression and the promoter DNA methylation Beta value was evaluated with Pearson’s correlation. *P* value < 0.05 was considered significant. R package survival was used to fit survival time and survival status within groups. Survival risk was estimated using cox-proportional-hazards model and statistical difference by log-rank test.

### Annotation of the tumor immune microenvironment

ESTIMATE algorithm was performed to evaluate the composition of tumor microenvironment by calculating the immune score (immune cell infiltration level) and stromal score (stromal content) for each sample. CIBERSORT algorithm was run to estimate the relative fraction of 22 immune cell types in tumor tissues (41). Spearman’s correlation coefficient was used to calculate the association between TIME features (immune cells’ fractions, immune and stromal scores, TMB, MSI and immune subtypes) and pyroptosis level. Association between PRGs expression and immune subtype was performed using the Kruskal-Wallis test.

### Drug sensitivity

The IC50 values of FDA-approved drugs determined in 60 human cancer cell lines (NCI60) and mRNA expression of these 60 cancer cell lines were obtained for estimation of correlation between drug IC50 and mRNA expression of PRGs using the Pearson’s correlation test. A negative correlation means that gene expression is suppressed indicating sensitivity to that drug and vice versa.

### Immunohistochemistry

Tumor types (COAD, HNSC, KIRC, LIHC, LUAD, LUSC) and PRGs (GSDMC, GSDMD, GSDME, NLRP3, NLRC4, IL1B) were selected for immunohistochemistry (IHC) based on their expression pattern in TCGA cancers, clinical relevance, tumor epidemiology, and sample availability. Lung cancer, colorectal cancer and liver cancer are predominant worldwide; head & neck cancer is predominant in Guangdong province of China; and kidney cancer was the most relevant in terms of PRGs expression and prognosis. Diagnosis was confirmed based on the histopathology of the patient’s tissue samples that were taken *via* biopsy before starting the treatment. Paraffin-embedded normal and tumor tissue sections were dewaxed in xylene and graded alcohol and boiled in sodium citrate buffer (pH 6.0) for antigen retrieval followed by blocking of endogenous HRP activity with 3% hydrogen peroxide. After washing with 10% phosphate buffered saline (PBS) and marked with PAP pen, the sections were blocked with 5% BSA and incubated with primary antibodies against GSDMC (Affinity Biosciences, #DF4157, Rabbit, 1:100), GSDMD (Proteintech, #20770-1-AP, Rabbit, 1:100), GSDME (Proteintech, #13075-1-AP, Rabbit, 1:100), NLRP3 (Proteintech, #19771-1-AP, Rabbit, 1:100), NLRC4 (Affinity Biosciences, #DF13673, Rabbit, 1:100), and IL1B (Proteintech, #16806-1-AP, Rabbit, 1:100) at 4 °C overnight. Next, the sections were incubated with a biotinylated goat anti-rabbit IgG secondary antibody for 20 min at room temperature and visualized with 3, 5- diaminobenzidine (DAB) Substrate Kit and finally counterstained with Hematoxylin. The staining intensity was scored using a semi-quantitative approach as follows: 0, negative; 1, weak; 2, moderate; and 3, strong. The frequency of positive cells was defined as follows: 0, less than 5%; 1, 5–25%; 2, 26–50%; 3, 51–75%; and 4, greater than 75%. The final IHC scores were obtained by multiplying the staining intensity and the frequency of positive cells. When tissue staining was heterogeneous, each area was scored independently and the scores of each area were added together as the final result. Patient informed consents were obtained and approval of the internal review and ethics boards of the Affiliated Cancer Hospital and Institute of Guangzhou Medical University was also acquired.

### Statistical analysis

The statistical software R v4.0.3 (http://www.r-project.org) was used to carry out all the statistical analysis. Student t test/Wilcoxon test and ANOVA/Kruskal-Wallis test was used to compare the two or more groups. Correlation was estimated using Spearman’s correlation test, and Pearson correlation test was employed in the case of drug sensitivity analysis. “Mann-Kendall Trend Test” was used to assess the trend of PRGs with cancer stage. Kaplan-Meier survival curves using log-rank tests were used for prognostic significance. Survival risk was estimated using cox proportional-hazards model and statistical difference with log-rank test. Statistical significance was set at p-value of less than 0.05.

## Results

### Aberrant expression and prognosis

Pyroptosis-regulated genes (PRGs), including *GSDMA, GSDMB, GSDMC, GSDMD, GSDME (DFNA5), PJVK (DFNB59), NLRP1, NLRP3, NLRP9, NLRC4, AIM2, CASP1, CASP4, CASP5, CASP6, CASP8, CASP11 (SCAF11), IL1B, IL18, ZBP1, PYCARD, GZMB, GZMA, PRF1, CPTP, DHX9, NAIP, DDX3X, APIP, CTSG* and *GPX4*, were identified based on the previous studies (1, 8, 42). In order to fully appreciate pyroptosis dysregulation, we first evaluated the pyroptosis level in normal tissue by obtaining PRGs’ expression in human normal tissues as illustrated in **Figure S1A**. A uniform normal gene expression outlook can be observed for the majority of the organs except for the brain, heart, skeletal muscle and pancreas. *GPX4, DDX3X, DHX9*, and *CPTP* showed overexpression across all normal tissues. To further explore the interaction of PRGs, gene-network and protein-protein interaction (PPI) analysis was conducted as shown in **Figures S1B-D**. A strong interaction network is evident among the core PRGs indicating a robust connectivity, which is also evident in the co-expression profile as shown in **Figure S1C**. Furthermore, gene ontology (GO) and REACTOME pathways enrichment analysis indicated involvement of PRGs in pyroptosis, inflammatory and immune-related pathways as illustrated in **Figures S2A, B**. After establishing the normal gene expression outlook, we proceeded with further pan-cancer gene differential expression analysis.

**Figure 1:**
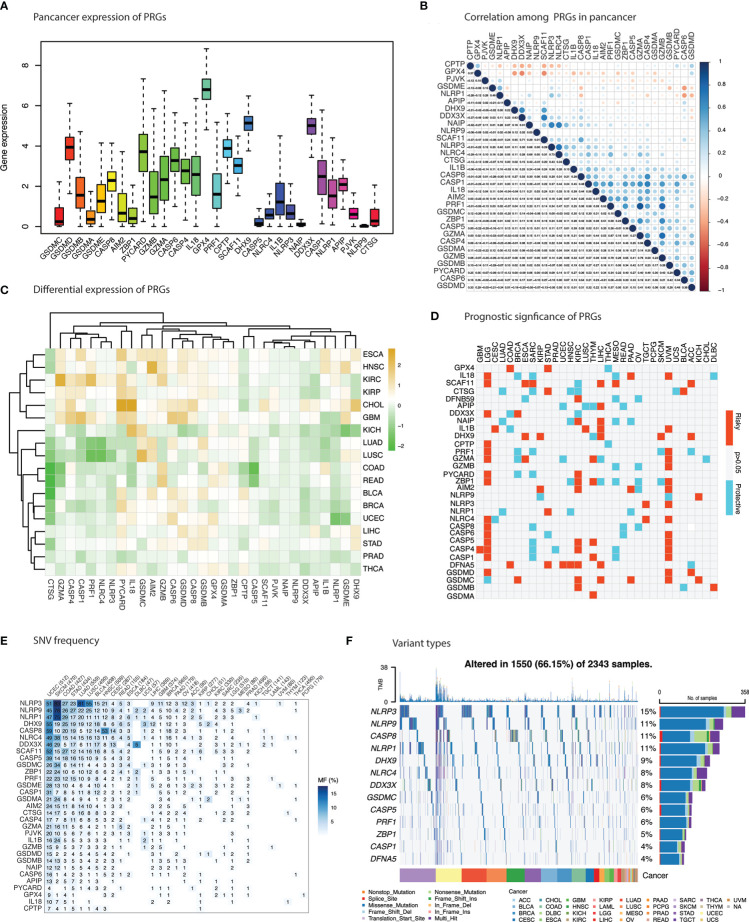
Differential expression, survival risk and mutations in pyroptosis-related genes (PRGs). **(A)** Cumulative expression of PRGs across cancers. **(B)** Correlation between PRGs in cancer. Blue indicate positive correlation and red indicate negative correlation. **(C)** Differential expression of PRGs in 18 cancers. Brown & green represent upregulation & downregulation of PRGs in cancers respectively. **(D)** Survival risk of PRGs. Red and blue indicate risky and protective effect of PRGs enrichment. **(E)** Histogram shows the frequency of single nucleotide variation for each PRG in each cancer type. **(F)** Oncoplot depicting top 10 mutated PRGs in cancers.

Overall pan-cancer expression of each gene is illustrated in **Figure 1A** indicating a strong pan-cancer expression of *GSDMD, PYCARD12, GPX4, DHX9* and *DDX3X*. Comparative expression levels for main gasdermins (*GSDMB, GSDME, AIM2*) and inflammasomes (*NLRP1, NLRP3*, and *NLRC4*) could also be observed. With the exception of *GSDME* and *NLRP1*, a strong positive correlation in the pan-cancer mRNA expression for these two group of genes is indicated in **Figure 1B**. Pan-cancer expression analysis unraveled significant dysregulated expression of PRGs in 18 types of cancers (**Figure 1C** and **Table S1**). Significantly upregulated PRGs included *PYCARD* in 12 cancers; *GSDMB* in 10 cancers; *IL18* in 9 cancers; *GSDMD, CASP8, GZMB*, and *GPX4* in 8 cancers; *AIM2* and *CASP6* in 7 cancers; *GZMA* in 6 cancers; *GSDME, CASP4* and *DHX9* in 5 cancers; *GSDMA* and *GSDMC* in 4 cancers (**Figure 1C**). *CTSG, NLRP9, DFNB59, APIP, SCAF11, CASP5* and *NAIP* showed significant downregulation across cancers. Certain cancer-specific expression patterns were observed. ESCA, HNSC, KIRC, KIRP, CHOL and GBM showed uniform upregulation pattern for the most PRGs except the aforementioned downregulated PRGs. KICH, LUAD, LUSC, COAD, and READ demonstrated a predominant suppressive pattern of PRGs’ expression. A third group of cancers comprised of BLCA, BRCA, UCEC, LIHC, STAD, PRAD and THCA showed a similar expression pattern with first group of cancers except for the contrast behavior of *NLRP3, NLRC4, PRF1, CASP1, CASP4*, and *GZMA* (downregulated in this group). *GSDME* demonstrated higher expression in LUSC, KIRP, and HNSC and lower expression in BRCA, UCEC, KICH, and PRAD. A similar but slightly contrast pattern of mixed expression was unraveled for *NLRP1, NLRP3*, and *NLRC4* with strong downregulation in UCEC, KICH (*NLRP1*), LUAD and LUSC (*NLRP3* and *NLRC4*) which constitute critical components of pyroptosis. Aberrant expression of essential PRGs such as, the Gasdermins and inflammasomes, is highly indicative of pyroptosis involvement in tumorigenesis.

Importantly, dynamics in PRGs expression were associated with poor prognosis in several cancers as shown in **Figure 1D** (**Table S2**). Upregulation of PRGs in KIRC, LGG, UVM, LIHC and THYM and downregulation in OV, LUAD, SARC, and MESO predicted poor survival in these cancers. PRGs with consistent enhanced expression and association with poor survival in cancers included *GSDMC* in BRCA, KIRC, LIHC, PAAD, UVM and KICH; *DFNA5 (GSDME)* in COAD, STAD, UCEC, HNSC, KIRC; and *AIM2* in KIRP, KIRC, UVM, and PAAD.

### Genomic alterations of PRGs in cancer

Analysis of single nucleotide variants (SNVs) were sought to identify its impact on gene expression and determine variants in these cancers. Frequency of SNVs were higher in most of the cancers that exhibited PRGs suppression such as SKCM, COAD, STAD, LUAD, and LUSC (**Figure 1E**). The frequency of SNVs in PRGs was 66.15% (1550 of 2343 samples) with missense mutations as the main SNV type (**Figure 1F**). Top mutated genes were *NLRP3* (15%), *NLRP9* (11%), *CASP8* (11%), *NLRP1* (11%), *DHX9* (9%), *NLRC4* (8%). Among gasdermins, only *GSDMC* (6%) and *GSDME* (4%) were among the top ten PRGs.

**Figure 2:**
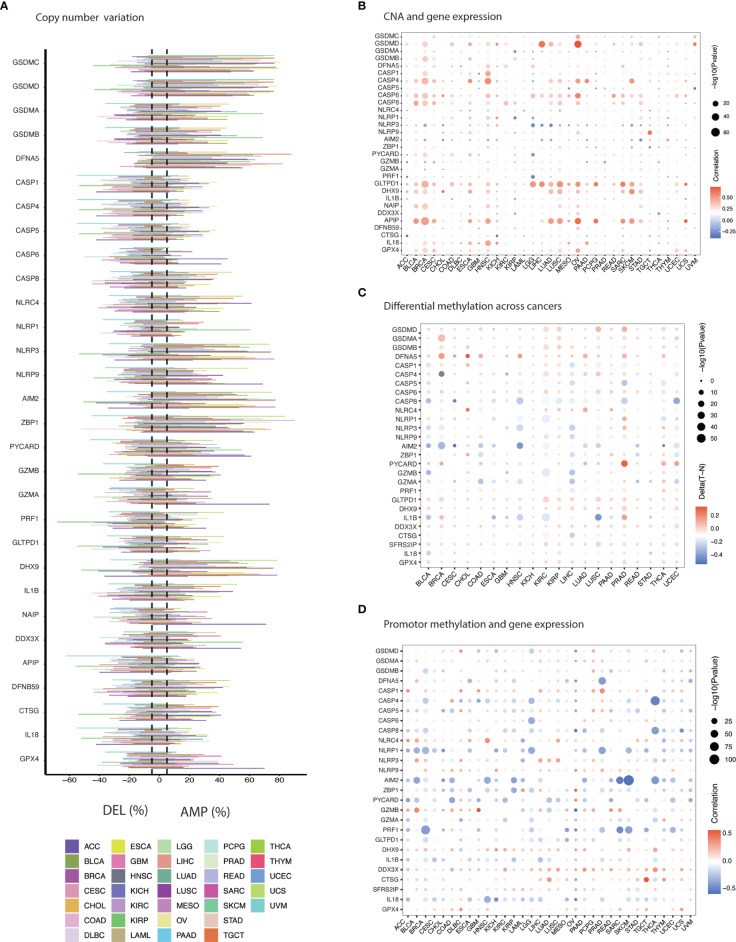
Genomic & epigenetic aberrations in PRGs. **(A)** Histogram illustrates frequency of copy number alteration (deletion/amplification) for each PRG across cancers. **(B)** The Spearman’s correlation between copy number variation (CNV) and the expression of PRGs. Blue & red represent negative & positive correlation respectively. **(C)** Heatmap shows the methylation difference between tumor and normal tissues. Red and blue indicate hypermethylation and hypomethylation in cancers, respectively. **(D)** Pearson’s correlation showing association between methylation and gene expression. Blue & red represent negative & positive correlation respectively.

Copy number variation (CNV) analysis unraveled a mixed profile for PRGs (**Figure 2A** and **Table S3**). *GSDMC, GSDMD, GSDME, ZBP1, AIM2, DHX9* and *NLRP3* demonstrated significant amplification across cancers (**Figure 2A**). Deletion was less common in these genes and can be observed predominantly in caspases (*CASP1, CASP4, CASP5, CASP6*), *PRF1, APIP*, and *IL1B*. Moreover, CNV showed a positive correlation with PRGs expression which was more common in OV, ESCA, HNSC, BLCA, BRCA, LUSC, STAD, and CESC among others as shown in **Figure 2B** (**Table S4**). *APIP, GLTPD1, CASP6, CASP4, GSDMD, CASP8, DHX9*, and *GPX4* were commonly correlated PRGs with CNV frequency. These results indicate that copy number aberrations in PRGs appears to influence their gene expression.

Gene expression can also be regulated by promoter methylation and aberrant methylation has been associated with tumorigenesis (43). Comparing the difference in methylation level between cancer and normal tissues revealed negative correlation for majority of the PRGs in several cancer tissues such as BLCA, KIRC, HNSCC, LIHC, LUSC, COAD, LUAD, and BRCA (**Figure 2C** and **Table S5**). Hypermethylation for PRGs was mainly observed in BRCA, CHOL, KICH, KIRC, KIRP, LUAD, LUSC, PRAD, and THCA. Hypomethylation can be noticed in BLCA, HNSC, and LIHC. Gasdermines (*GSDMD, GSDMA, GSDMB*, and *GSDME*) showed hypermethylation across cancers. Hypomethylation across cancers was observed for *AIM2, CASP8, GZMA*, and *IL-1B*. A majorly pan-cancer positive correlation between promotor methylation and gene expression of *GSDMA, CASP1, NLRP9, GZMB, DHX9, DDX3X, SFRS2IP (CASP11)* and *GPX4* was observed (**Figure 2D** and **Table S6**). An inverse relation (negative correlation) for other PRGs was evident such as *CASP8, NLRP1, AIM2, ZBP1, PYCARD, PRF1, ILIB*, and *IL18*. Overall, a robust association was apparent between methylation and gene expression.

**Figure 3:**
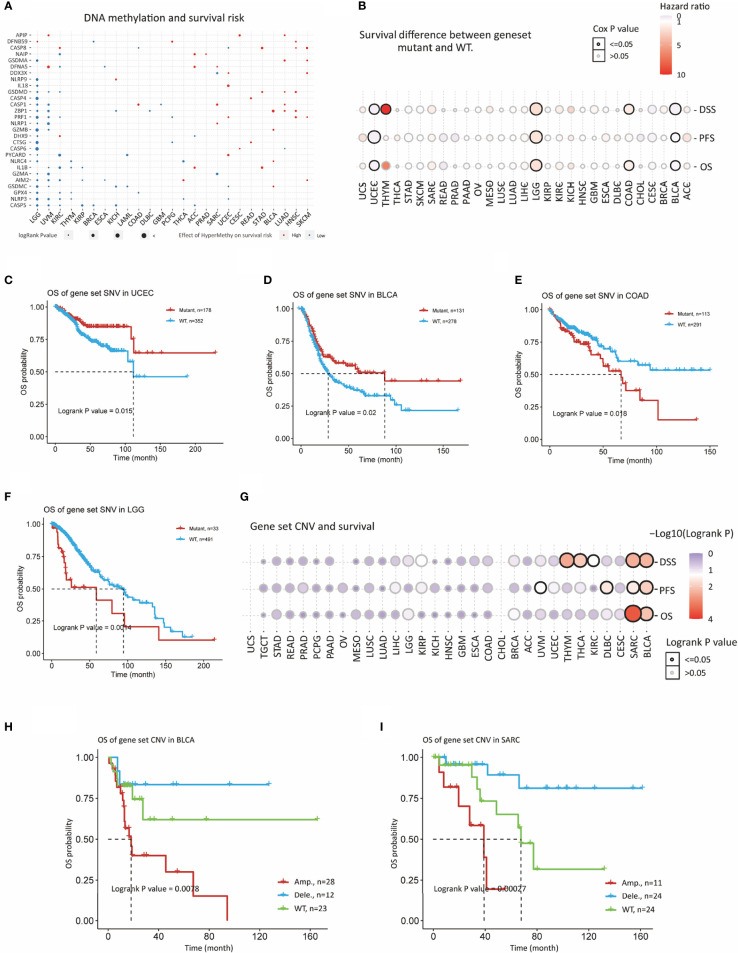
Prognostic significance of genomic aberrations in PRGs. **(A)** Survival risk of methylation of PRGs. Red and blue indicate risky and protective effect of PRGs methylation. **(B)** Bubble plot depicts survival difference between SNV mutant and WT groups in cancers. **(C)** Kaplan-Meier curves shows survival difference between SNV mutant and WT groups in UCEC, **(D)** BLCA, **(E)** COAD, and **(F)** LGG **(G)** Bubble plot depicts survival difference between CNV Amp, Del, and WT in cancers. **(H)** Kaplan-Meier curves survival difference between CNV Amp, Del, and WT in BLCA and **(I)** SARC.

Survival significance of genomic aberrations was also considered. Methylation showed significant association with prognosis of various cancers (**Figure 3A** and **Table S7**). For example, methylation of PRGs in LGG, UVM and KIRC were predictive of improved survival as PRGs are risky in these cancers. While methylation of some of the PRGs showed increased survival risk in cancers like SKCM, HNSC, LUAD, STAD, and READ. Methylation of *GPX4, NLRP3*, and *CASP5* were associated with better prognosis in majority of cancers. In case of genomic variation, we evaluated the influence of gene set SNV on prognosis (**Figure 3B** and **Table S8**). UCEC and BLCA patients with mutant gene set SNV had better survival as compared to WT (wild-type) as illustrated in **Figures 3C, D**. A contrast result was evident for COAD and LGG (**Figure 3B**). Gene set CNV also had significant impact on OS, PFS and DSS in SARC and BLCA (**Figures 3B, E, F** and **Table S9**). Amplification of gene set CNV was predictive of worst prognosis as compared to WT and deletion as shown in **Figures 3G-I**). As evident from these results, genome variance appears to influence gene expression of PRGs and prognosis in slected cancers indicating great functional implications.

### Pyroptosis level and clinical implications

**Figure 4:**
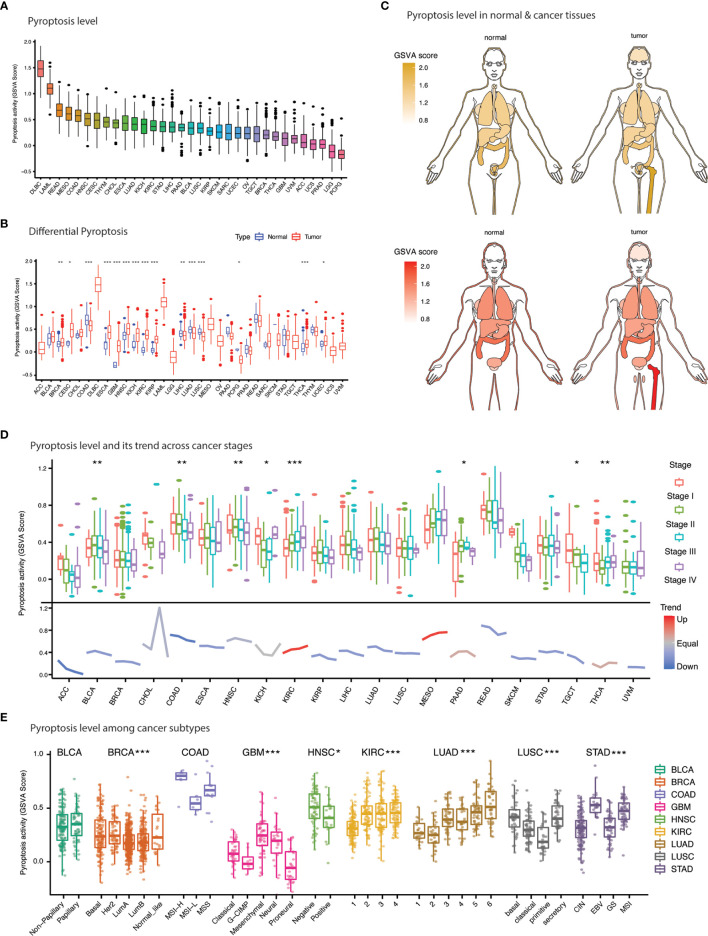
Pyroptosis level and clinical features. **(A)** Anatogram depicting the differential pyroptosis level (GSVA score) between normal (left) and cancer (right) tissues. Yellow and red represent pyroptosis level in female and males. **(B)** Bar plot illustrates the pyroptosis level (GSVA score) across cancers. **(C)** Comparison of pyroptosis level (GSVA score) between normal and cancer tissues across 33 cancers. **(D)** Pyroptosis level and its tendency with cancer stages. **(E)** Pyroptosis level association with cancer subtypes. * *p* value < 0.05; ** *p* value < 0.01; *** *p* value < 0.001.

Gene set variation analysis (GSVA) computation yielded GSVA scores, which is described as pyroptosis level, for 33 types of cancers and adjacent-normal tissues (**Figures 4A-C** and. **Table S10**). Overall, pyroptosis level was the highest DLBC, LAML, READ, and MESO and the lowest in PCPG, LGG, PRAD, UCS, and ACC (**Figure 4A**). The difference in pyroptosis level was significant for 15 types of cancers including BRCA, CESC, COAD, ESCA, GBM, HNSC, KICH, KIRC, KIRP, LUSC, LUAD, LIHC, PCPG, THCA, and UCEC (**Figures 4B, C**). Pyroptosis level was significantly higher in cancers compared to normal tissues for 10 cancers (BRCA, CESC, COAD, ESCA, GBM, HNSC, KICH, KIRC, KIRP, THCA, and UCEC). Suppression of pyroptosis was evident in 5 cancers including COAD, LUSC, LUAD, LIHC, and PCPG.

We further evaluated the impact of pyroptosis level on tumor characteristics such as pathological stage and molecular subtypes. Significant differences in pyroptosis level were evident among 8 cancers (**Figure 4D**). A remarkable downward trend was observed for most of the cancers except KIRC and MESO suggesting that the PRGs expression is progressively downregulated with disease progression (**Figure 4D**). KIRC which exhibit strong upregulation of PRGs; however, showed a contrast opposite trend. Moreover, molecular subtypes of various cancers also exhibited incredible heterogeneity (**Figure 4E**). For example, a significantly lower pyroptosis level was achieved for luminal subtype as compared to other subtypes in BRCA. In fact, each molecular subtype of COAD, GBM, HNSC, LUAD, LUSC, and STAD demonstrated a distinct pyroptosis level. These results indicate that pyroptosis varies with the molecular make-up of the cancers.

**Figure 5:**
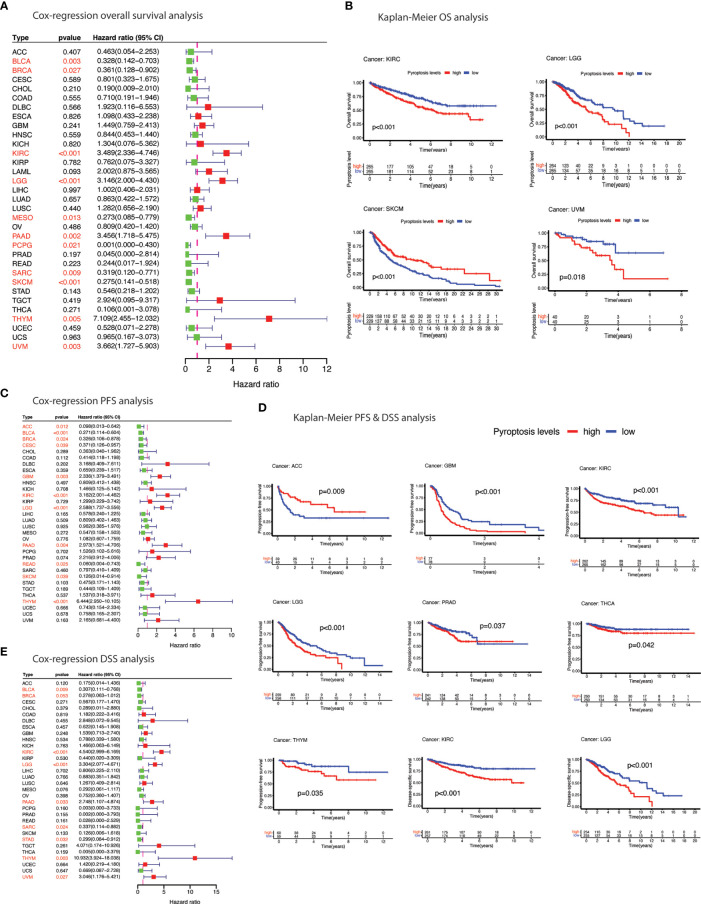
Prognostic significance of pyroptosis in cancer. **(A)** Forest plot of cox-regression overall survival (OS) difference between pyroptosis-low and pyroptosis-high groups. **(B)** Kaplan-Meier curves depicting overall survival (OS) difference between pyroptosis-low and pyroptosis-high groups. **(C)** Forest plot of cox-regression progression-free survival (PFS) difference between pyroptosis-low and pyroptosis-high groups. **(D)** Kaplan-Meier curves depicting progression-free survival (PFS) & disease-specific survival (DSS) difference between pyroptosis-low and pyroptosis-high groups. **(E)** Forest plot of cox-regression disease-specific survival (DSS) difference between pyroptosis-low and pyroptosis-high groups. Red color indicates significant survival difference (p<0.05).

Prognostic relevance of pyroptosis was sought by undertaking survival analysis using TCGA survival data. Higher pyroptosis level was associated with worst prognosis in majority of the cancers in terms of OS (KIRC, LGG, and UVM), PFS (GBM, KIRC, LGG, PRAD, THCA, and THYM) and DSS (KIRC and LGG) as estimated by Kaplan-Meier curves (**Figures 5B, D** and **Table S11-13**). Protective role of the pyroptosis was also evident in several cancers based on the cox-regression survival analysis (**Figures 5A, C, E**). For example, BLCA, BRCA, PAAD, SARC, SKCM and THYM showed better survival outcomes (OS, PFS, DSS) in patients with higher pyroptosis level.

### Immunological landscape

**Figure 6:**
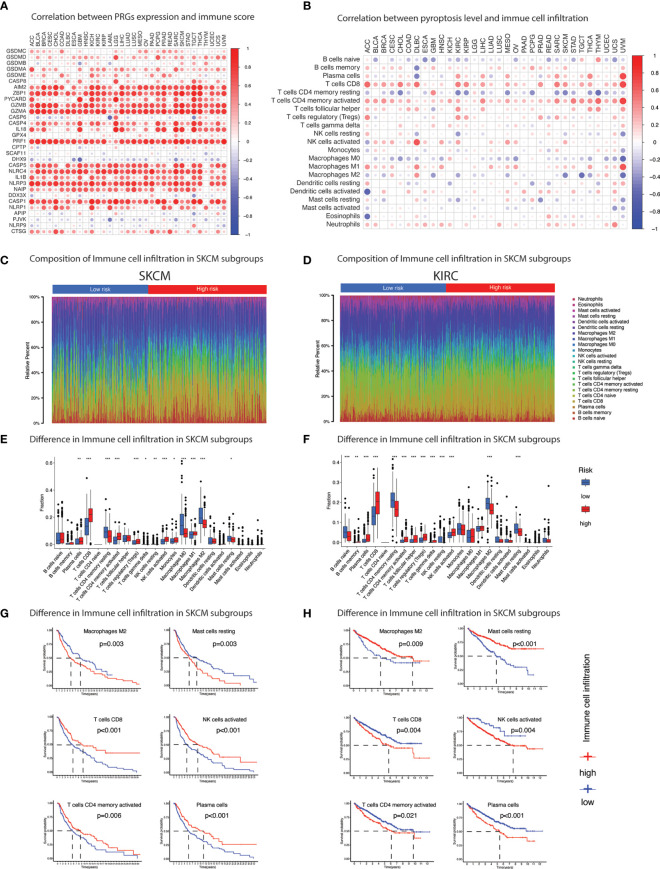
Immune cell infiltration. **(A)** Heatmap summarizes spearman correlation between pyroptosis level and ESTIMATE immune score. Red & blue indicate positive & negative correlation. **(B)** Heatmap summarizes spearman correlation between pyroptosis level and infiltration of 22 types of immune cells estimated by CIBERSORT algorithm. Red & blue indicate positive & negative correlation. **(C-F)** Pyroptosis subgroups (pyroptosis-low and pyroptosis-high) based on median pyroptosis level & proportions of TME cells in SKCM (C & E) and KIRC (D & F). The thick lines represent the median value. The bottom and top of the boxes are the 25th and 75th percentiles (interquartile range), respectively. Significant statistical differences between the two subgroups were assessed using the Wilcoxon test. * *p* value < 0.05; ** *p* value < 0.01; *** *p* value < 0.001; (*p* value > 0.05). **(G)** Kaplan-Meier curves of survival analysis of immune cells in SKCM and **(H)** KIRC based on the infiltration level.

**Figure 7:**
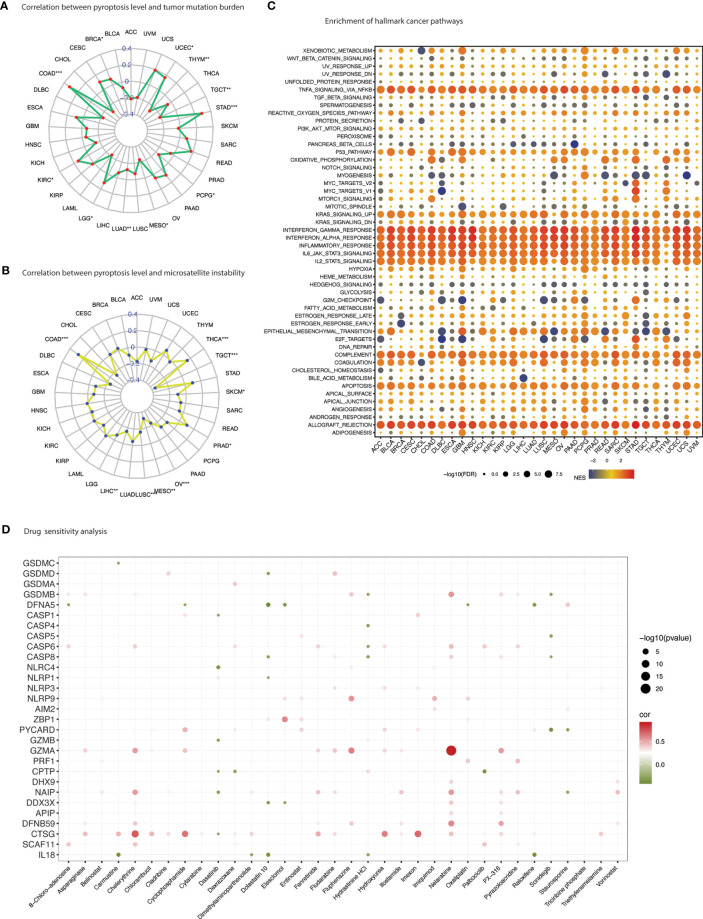
Therapeutic vulnerabilities. **(A)** Spider web plot summarizes spearman correlation between pyroptosis level and tumor mutation burden. * *p* value < 0.05; ** *p* value < 0.01; *** *p* value < 0.001. **(B)** Spider web plot summarizes spearman correlation between pyroptosis level and microsatellite instability. * *p* value < 0.05; ** *p* value < 0.01; *** *p* value < 0.001. **(C)** Enrichment analysis for hallmark cancer pathways between pyroptosis-high and pyroptosis-low tumor tissues. NES is the normalized enrichment score in the GSEA algorithm. **(D)** Drug sensitivity analysis of PRGs based on the CellMiner database. Green depicts negative correlation (sensitivity to drug) and red shows positive correlation (induction of gene expression).

Pyroptosis has gained tremendous scientific attention for its close association with modulation of immune responses. Individually PRGs demonstrated a positive correlation with immune score which was comparatively more visible in inflammasomes (*AIM2, NLRP3, NLRP1, NLRC4*) than gasdermins (*GSDMA, GSDMB, GSDMC, GSDMD*) (**Figure 6A** and **Table S14**). A similar but less prominent association was also evident for stromal content (**Figure S2C** and **Table S15**). Overall, pyroptosis enrichment was more positively correlated with immune score than stromal score as illustrated in **Figure S3A**. Dissecting immune composition of the tumor microenvironment demonstrated an extraordinary positive correlation, that was consistent across tumors, between pyroptosis level and infiltration of major T cell subtypes (**Figure 6B**). High pyroptosis level was indicative of high infiltration of key immune cells such as CD8+ T cells, activated CD4 memory T cells, follicular helper T cells, regulatory T cells, Gamma delta T cells, memory B cells, activated NK cells, M1 macrophages, activated dendritic cells, and neutrophils (**Figure 6B**). In contrast, naïve B cells, resting CD4 memory T cells, and M0 macrophages observed strong negative correlation. In fact, the pyroptosis association with immune cell infiltration was similar regardless of the prognosis as illustrated in **Figures 6C-H**. The association of immune cells with pyroptosis level in SKCM and KIRC (two cancers exhibiting contrast prognosis with enrichment of pyroptosis) was maintained even though the infiltration of immune cells had the opposite impact on prognosis (**Table S16**). For example, infiltration of CD8+ T cells showed positive correlation with pyroptosis enrichment in both cancers but was associated with poor prognosis in SKCM and vice versa (**Figures 6D-H** and **Table S16**). Nonetheless, a hot immune microenvironment is indicated which suggests suitability for intervention with immune checkpoint blockade. Besides, individual PRGs and pyroptosis level also showed strong association with the IFN-γ dominant type (immune subtype 2/C2) (**Figures S2D, E**). Pyroptosis was comparatively enriched in the C2-categorized patients of all 33 TCGA cancer types except ESCA and PCPG (**Figure S2E**). In the context of immunotherapy, pyroptosis level also showed positive correlation with immunotherapy biomarkers, tumor mutation burden (TMB) and microsatellite instability (MSI) (**Figures 7A, B** and **Table S17-18**). TMB was positively associated with pyroptosis in BRCA, COAD, KIRC, LGG, STAD and UCEC while MSI showed positive correlation with COAD, PRAD, and THCA. Overall, these outcomes indicate that patients with positive enrichment of pyroptosis pathways may response to immunotherapy. Alternatively, induction of this pathway can certainly generate hot immune microenvironment for therapeutic exploration.

### Cancer pathways

In coherence with the individual PRGs activity in cancer pathways, pyroptosis level also showed close association with inflammatory and immune-related pathways (**Figure 7C**). Additionally, several oncogenic pathways were also enriched with high pyroptosis level, including P53 pathway, DNA repair, KRAS signaling, epithelial-mesenchymal transition, IL6 JAK STAT3 signaling, IL2 STAT5 signaling, PI3K AKT MTOR signaling and angiogenesis (**Table S19**). Certain metabolism-related hallmarks were also positively correlated with pyroptosis level such as xenobiotic metabolism, heme metabolism, fatty acid metabolism, estrogen response (early & late), cholesterol homeostasis and adipogenesis. Further metabolic activity was unraveled using the KEGG metabolism-related pathways enrichment analysis which indicated high enrichment in several fatty acids, amino acids, and nucleotides metabolism, Type-I diabetes, oxidative phosphorylation, and production of ribosome and proteasome (**Figure S3B** and **Table S20**).

### Drug sensitivity

Drug sensitivity analysis represents an essential component of genomic investigations to identify clinical vulnerabilities. Drug-sensitivity of PRGs was sought through correlating drug sensitivity and pyroptosis gene expression (PRGs) profiling data across various cancer cell lines (**Figure 7D** and **Table S21**). *GSDME* showed sensitivity to a number of drugs including cyclophosphamide, dolastatin 10, elesclomol, oxaliplatin and raloxifene; *GSDMC* to carmustine; *GSDMD* to dolastatin 10; *GSDMB* to hydrastinine HCL and sonidegib; *NLRC4* to dasatinib; and *NLRP1* to dolastatin 10. Chemotherapy drugs such as nelarabine, fludarabine and oxaliplatin were positively correlated with PRGs expression including *GSDMB* and *NLRP3*. Other drugs that showed positive correlation included PX-316 (an AKT inhibitor), fenretinide (a synthetic analog of the naturally occurring retinol (vitamin A)), and fluphenazine (a typical antipsychotic).

### Validation of PRGs expression in clinical specimens

**Figure 8:**
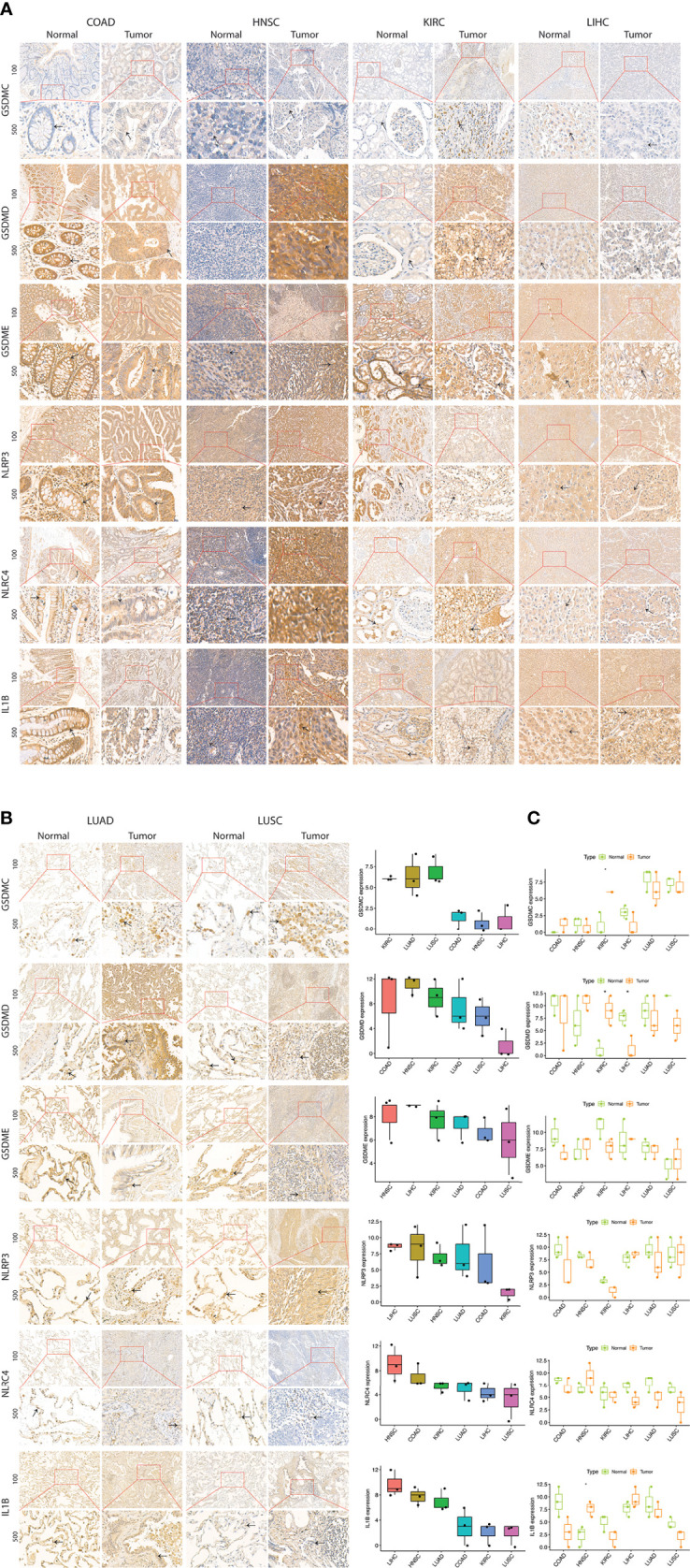
Expression of selected PRGs in clinical samples. **(A)** Representative images of expression (brown, cell cytoplasmic/nucleus stain) of GSDMC, GSDMD, GSDME, NLRP3, NLRC4, and IL1B in the clinical samples of colon adenocarcinoma (COAD), head & neck squamous cell carcinoma (HNSC), kidney renal cell carcinoma (KIRC), liver hepatocellular carcinoma (LIHC), lung adenocarcinoma (LUAD), and lung squamous cell carcinoma (LUSC). **(B)** Expression level (IHC quantification) of PRGs in the clinical samples of colon adenocarcinoma (COAD), head &. Neck squamous cell carcinoma (HNSC), and kidney renal cell carcinoma (KIRC), liver hepatocellular carcinoma (LIHC), lung adenocarcinoma (LUAD), and lung squamous cell carcinoma (LUSC). **(C)** Differential expression (IHC quantification) of PRGs in the clinical samples of colon adenocarcinoma (COAD), head &. Neck squamous cell carcinoma (HNSC), and kidney renal cell carcinoma (KIRC), liver hepatocellular carcinoma (LIHC), lung adenocarcinoma (LUAD), and lung squamous cell carcinoma (LUSC). P values are shown as: *P<0.05; **P < 0.01; ***P < 0.001 (t-test).

The specific tumors (COAD, HNSC, KIRC, LIHC, LUAD, LUSC) were selected for histopathology validation based on their expression pattern (as reported in results subsection aberrant expression and prognosis), tumor epidemiology and clinical relevance (see method section for details). The expression of selected 6 PRGs (GSDMC, GSDMD, GSDME, NLRP3, NLRC4, and IL1B) was assessed in 6 types of cancers and adjacent normal tissues (**Figures 8A-C**). A dysregulated expression profile was apparent for these 6 PRGs across cancers mainly observed in cytosol. In addition to the significantly higher expression in KIRC, GSMDC and GSDMD were also significantly suppressed in LIHC, respectively. GSDME demonstrated a comparatively high expression across cancers, which was mainly found near nuclear envelope, but showed no significant differential elevation. NLRP3 was detected at low level in KIRC but overexpressed in LUAD and LUSC. IL1B was significantly overexpressed in HNSC. Overall, the expression profile of PRGs shows involvement of this pathway in cancer development which warrants further exploration in larger studies.

## Discussion

Pyroptosis is considered to play both anti and pro-cancer roles in cancer. Pan-cancer probe of pyroptosis is essential in order to apprehend its “double-edged sword character” in cancer. We have reported a detailed account of 31 pyroptosis regulated genes across 33 types of cancers by carrying out multi-omics data mining investigations. Gene expression and genomic alterations as well as prognostic significance of PRGs were accounted for. Moreover, immune characteristics and therapeutic vulnerabilities were also determined.

Cancer initiation, development, progression and metastasis originate from genetic variations and epigenetic alterations (44). Genetic and epigenetic abnormalities were apparent in driving the diverse PRGs’ expression, immune microenvironment dynamics, and cancer prognosis. DNA methylation is one of the epigenetic mechanisms implicated in gene expression control (45). Hypermethylation as well as hypomethylation has been described in carcinogenesis (44–46). DNA methylation level of PRGs was significantly higher in tumors compared to paired-adjacent normal tissues; associated with gene expression; and survival risk. We noticed three cancers (UVM, LGG and KIRC) that showed enhanced PRGs expression associated with a worst prognosis. Interestingly, PRGs had uniform hypomethylation in these cancers that was significant for poor survival. Previous studies have also associated DNA hypomethylation with tumor progression or the level of malignancy (47, 48). PRGs may undergo active demethylation, at least in part, in these three cancers during tumor progression, and targeting this pathway may yield a better prognosis. On the other hand, tumors such as SARC and SKCM, which undergo significant CNV and SNV alterations respectively, showed a contrast outcome. In these two cancers, patients with high level of pyroptosis were associated with better prognosis compared to patients with low pyroptosis. Both of these genomic variations have been extensively studied for their effects on gene expression and functional implications (49, 50). Further elucidation is need to determine the underlying mechanistic details of the impact of genomic variation on PRGs’ expression and functionality. These outcomes suggest that PRGs are subjected to different genomic/epigenomic irregularities in various cancers that may determine their expression and prognosis.

Pyroptosis has been closely studied for inducing immunogenic responses in several cancers (51). Immune cell infiltration outlook was almost similar across cancers and clearly manifested pyroptosis as a bridge between innate and adaptive immunity (51). Overall cells from both components of the immune system were infiltrated based on the pyroptosis level and a hot tumor immune microenvironment was evident indicating vulnerability for immunotherapy. Exploration of tumors with significant difference in prognosis based on pyroptosis level revealed that pyroptosis level was driving the immune microenvironment regardless of the prognostic implications. As previously described, the only difference in these cancers was the type of genomic alteration in the PRGs which may hold prognostic significance. It appears genomic aberrances regulate PRGs expression causing functional alterations which may determine the overall immune response and cancer prognosis (49, 50). Nonetheless, pyroptosis show to correlate with various immune biomarkers and may be targeted with immunotherapy. On the other hand, induction strategies may also turn the cancer immune microenvironment into a hot one (52, 53). Further studies would be required to fully elucidate on these results in the future.

Pyroptosis is initiated and mediated by a variety of factors and players that yields in distinct pyroptotic pathways. Consequently, despite of overall gene set expression and its clinical significance, componential analysis of PRGs is also essential. NLRs and gasdermins, which constitute the main actors of pyroptosis, were also evident individually in their dysregulated gene expression, genomic aberrations, and clinical significance. In fact, *GSDMC, GSDMA, GSDMD*, and *AIM2* were the most upregulated genes across cancers. The highest SNV mutation frequency among PRGs was observed in *NLRP3, NLRP9, NLRP1, NLRC4, GSDMC*, and *GSDMA*. *AIM2* and *NLRP3* observed significant pan-cancer DNA methylation increase that was negatively correlated with gene expression. Moreover, hypomethylation of these two genes was associated with a worst outcome in various cancers. Hence, the identification of individual PRG paradigm is of great importance in devising strategies to target this pathway in selected cancers.

Having established a strong association between pyroptosis and carcinogenesis, the mechanism by which pyroptosis regulate cancer development and progression is still largely unclear. PRGs were unraveled to activate certain pathways including apoptosis, epithelial-mesenchymal transition (EMT) and estrogen receptor (ER) pathways among others. Activation of apoptotic pathway is evident by the fact that apoptosis and pyroptosis share some features and common mediators such as caspases (1, 2, 7). The association of pyroptosis and EMT is suggestive of its role in cancer invasion and metastatic tendencies. A recent multi-omics study with experimental validation has proved activation of GSDME for inflammatory pyroptosis by EMT-activating transcription factors ZEB1/2 *via* binding to GSDME gene promoter (54). ER pathway has also been involved in tumorigenesis and promote protumor TME particularly in hormone-sensitive tumors such as breast, ovarian, endometrial, and prostate (55). Inflammasome activation by activated fatty acids and high glucose levels observed in obesity, which has been linked to breast cancer and colorectal cancer, has also been acknowledged for obesity-associated cancer development (56, 57). NLRP3 inflammasome activation induced by E2 can also trigger pyroptosis and inhibit autophagy in HCC cells (58). A contrast outcome, inhibiting pyroptosis and activating autophagy, was unraveled in preventing atherosclerosis with estrogen (59). Moreover, estrogen as a treatment strategy in sepsis has been shown to suppress stress-induced pyroptosis (60). Hence, an interplay exists between these two pathways in inducing immunosuppression and carcinogenesis and can be targeted for therapeutic strategies.

Induction of pyroptosis has been highly anticipated as a therapeutic strategy. In fact, anti-cancer therapies such as targeted therapy, chemotherapy, and radiation therapy have been appreciated for their effects *via* induction of pyroptosis. Identification of therapeutic agents that can induce pyroptosis is vital. GSDME was shown to be sensitive to a number of anti-neoplastic drugs among others, which is reported to be suppressed *via* epigenetic inactivation in various cancers and considered as a tumor suppressor gene (34, 35, 61, 62). In fact, chemotherapy and targeted agents also exert their anti-tumor activity through GSDME-induced pyroptosis (63). Chemotherapy agents such 5-FU, paclitaxel and cisplatin were shown to induce GSDME-mediated pyroptosis in a caspase-3-dependent manner in gastric cancer and lung cancer, which is considered as a switch between apoptosis and pyroptosis (63–65). Inducing pyroptosis has also been appreciated for sensitizing cancers to other anti-neoplastic treatments such as chemotherapy and inducing potent anti-cancer immune responses. Targeted agent like PLK1 inhibitor has shown to sensitize cancer cells to cisplatin by inducing pyroptosis in oesophageal squamous cell carcinoma (66). The combination of BRAF and MEK inhibitors was shown to induce pyroptosis in melanoma cells *via* GSDME which resulted in an increase in CD4+ T cell and CD8+ T cell infiltration and a decrease in myeloid-derived suppressor cells (MDSCs) and tumor-associated macrophages (TAMs) (67, 68). Other gasdermins, GSDMB, GSDMC, GSDMD have also been extensively explored in cancers for their dichotomous behavior and consequently could be modulated with drugs identified in our study such as dolastatin 10. Identification of these anti-cancer drugs could also help in ushering certain pyroptotic mechanistic details.

Pyroptosis is mainly executed at protein-level and we have used TCGA data that only provide RNA-level quantification, which limits our study and may lead to certain inaccuracies. Nonetheless, our study validated protein level differential expression of these PRGs in selected cancers further strengthening the notion of dysregulation of pyroptosis in cancer. So far, only positive regulators are identified which has enabled us to assess fluctuations of pyroptosis level only. Nonetheless, negative regulators must also be identified to rectify the actual role of pyroptosis in cancer by constituting a pyroptotic-prognostic index. Moreover, lack of *in vitro* validation also limits the outcome of our study.

## Conclusions

In this study we have presented a comprehensive account of pyroptosis and pyroptosis-regulated genes across cancers. PRGs are differentially expressed in cancers and greatly influenced by the type of genomic aberrances. Not only gene expression but prognostic significance is also modulated by the type of genetic aberrances. Overall, genomic aberrations appear to drive diverse PRGs expression patterns and determine their functional implication in cancer. Pyroptosis was related to a hot tumor microenvironment regardless of the its association with prognosis. Moreover, several associated pathways and targeting agents are identified that may help in understanding and further exploration of mechanistic details of pyroptosis in cancer with a focus on therapeutic implications.

## Data availability statement

The original contributions presented in the study are included in the article/**Supplementary Material**. Further inquiries can be directed to the corresponding authors.

## Ethics statement

The studies involving human participants were reviewed and approved by Internal review and ethics boards of the Affiliated Cancer Hospital and Institute of Guangzhou Medical University. Written informed consent to participate in this study was provided by the participants’ legal guardian/next of kin.

## Author contributions

MK, MA, and KD conceived the experiment and performed data analysis. MK, MA, and JS prepared the pathology sections and performed the experiments. MK wrote the initial transcript. All authors have made significant contributions to the conception, supervision, and final approval of the manuscript.
