# Supplementary material for: Pyroptosis relates to tumor microenvironment remodeling and prognosis: A pan-cancer perspective
Source: Front Immunol. 2022 Dec 20;13:1062225. doi: 10.3389/fimmu.2022.1062225 (PMC9808401; doi:10.3389/fimmu.2022.1062225)
Supplement: Supplementary file 1 [file DataSheet_1.docx]

Pyroptosis relates to tumor microenvironment remodeling and prognosis: A pan-cancer perspective

**Supplementary Figures**


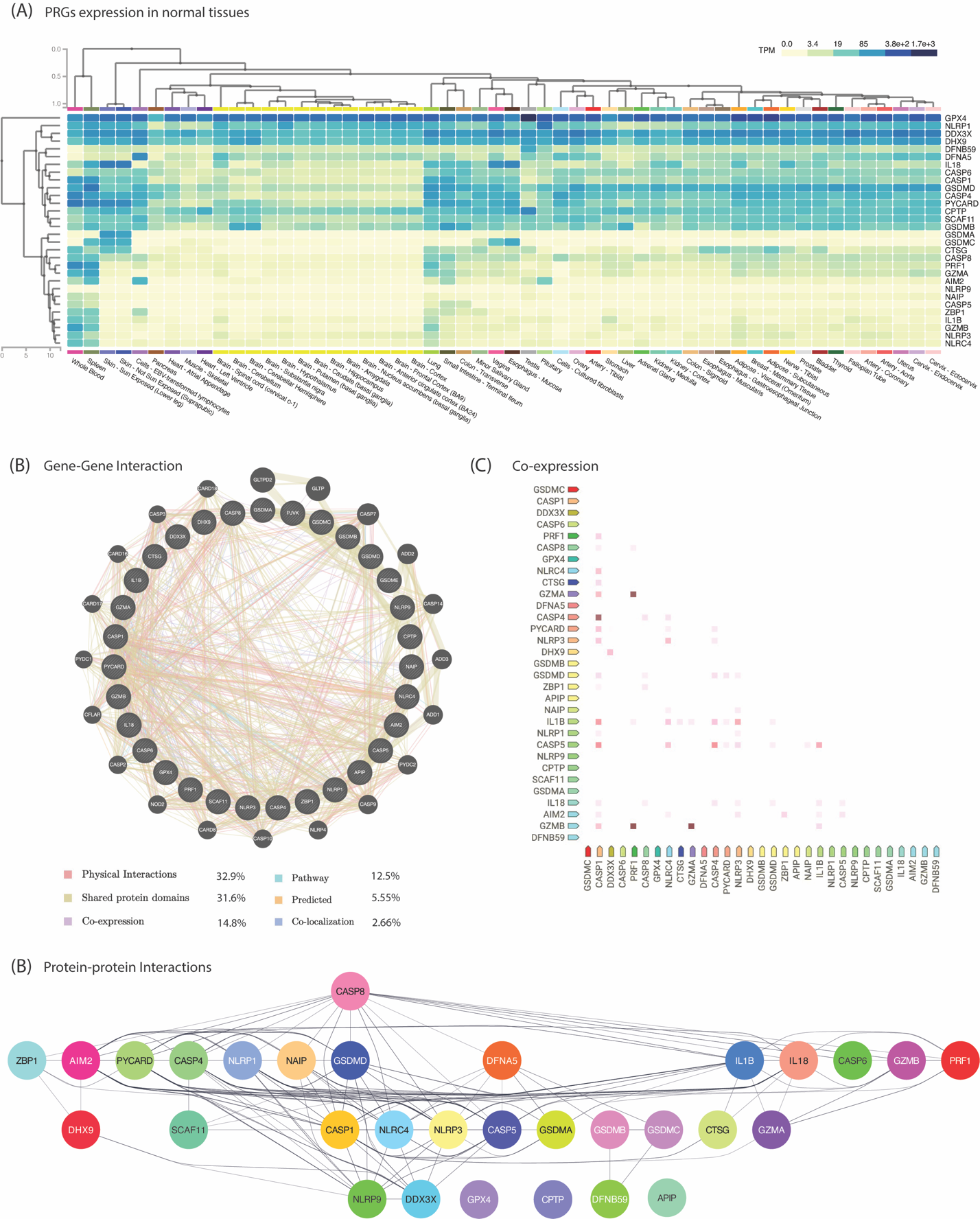


**Supplementary Figure 1. Pyroptosis in normal tissues**

(A) Heatmap illustrates the expression level of PRGs in normal tissues

(B) Network shows the gene-gene interactions among PRGs. The colors indicate the type of interaction:

(C) Co-expression of PRGs

(D) Protein-protein interactions among pyroptosis-related proteins.


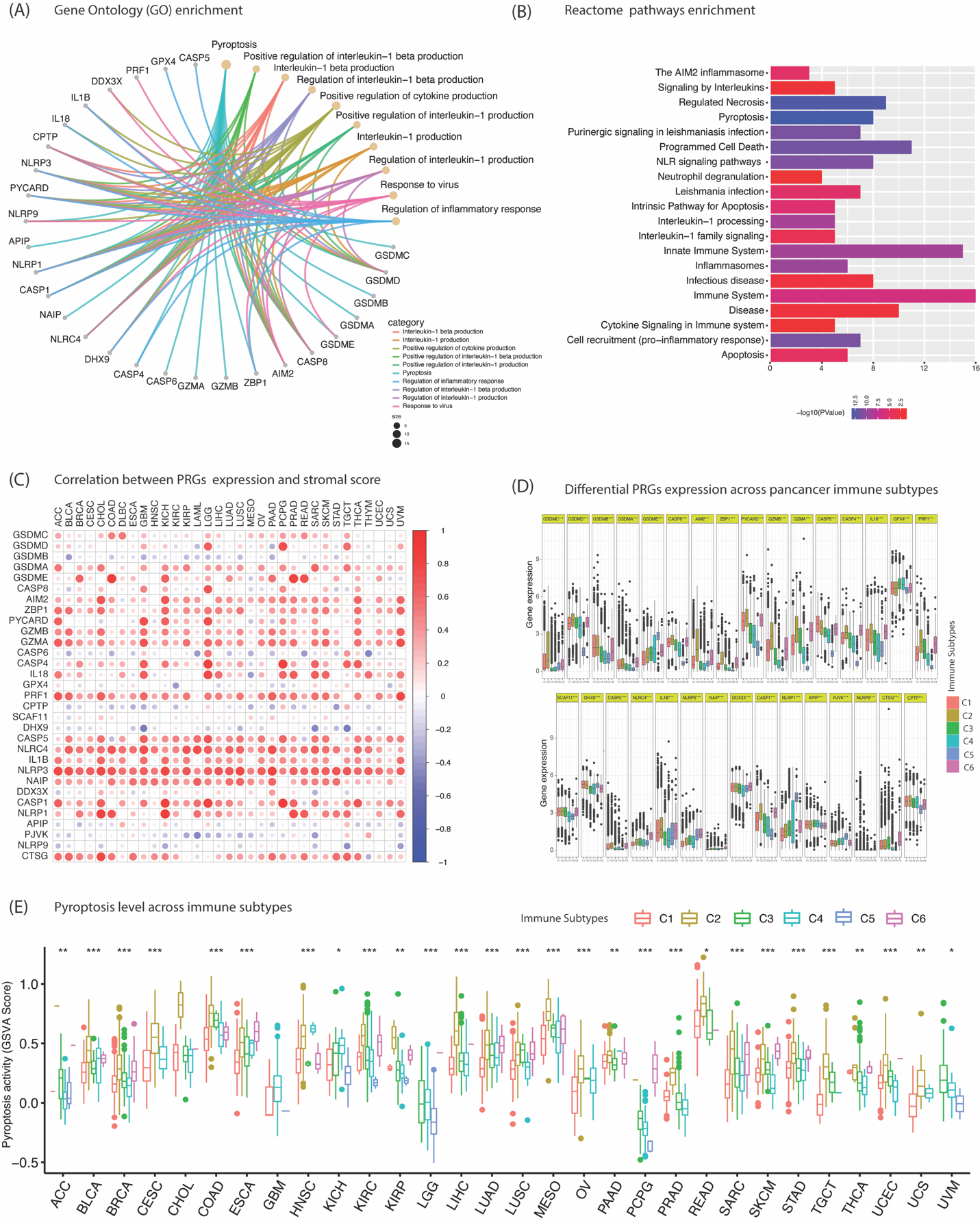


**Supplementary Figure 2. Enrichment analysis and immune features.**

(A) Gene Ontology (GO) enrichment analysis of PRGs.

(B) REACTOME pathways enrichment analysis of PRGs.

(C) Heatmap illustrating spearman correlation between pyroptosis level and ESTIMATE stromal score. Red & blue indicate positive & negative correlation.

(D) Bar plots of individual PRGs expression across TCGA immune subtypes. * *p* value < 0.05; ** *p* value < 0.01; *** *p* value < 0.001.

(D) Bar plots of pyroptosis level (GSVVA score) across immune subtypes of TCGA cancers. * *p* value < 0.05; ** *p* value < 0.01; *** *p* value < 0.001.


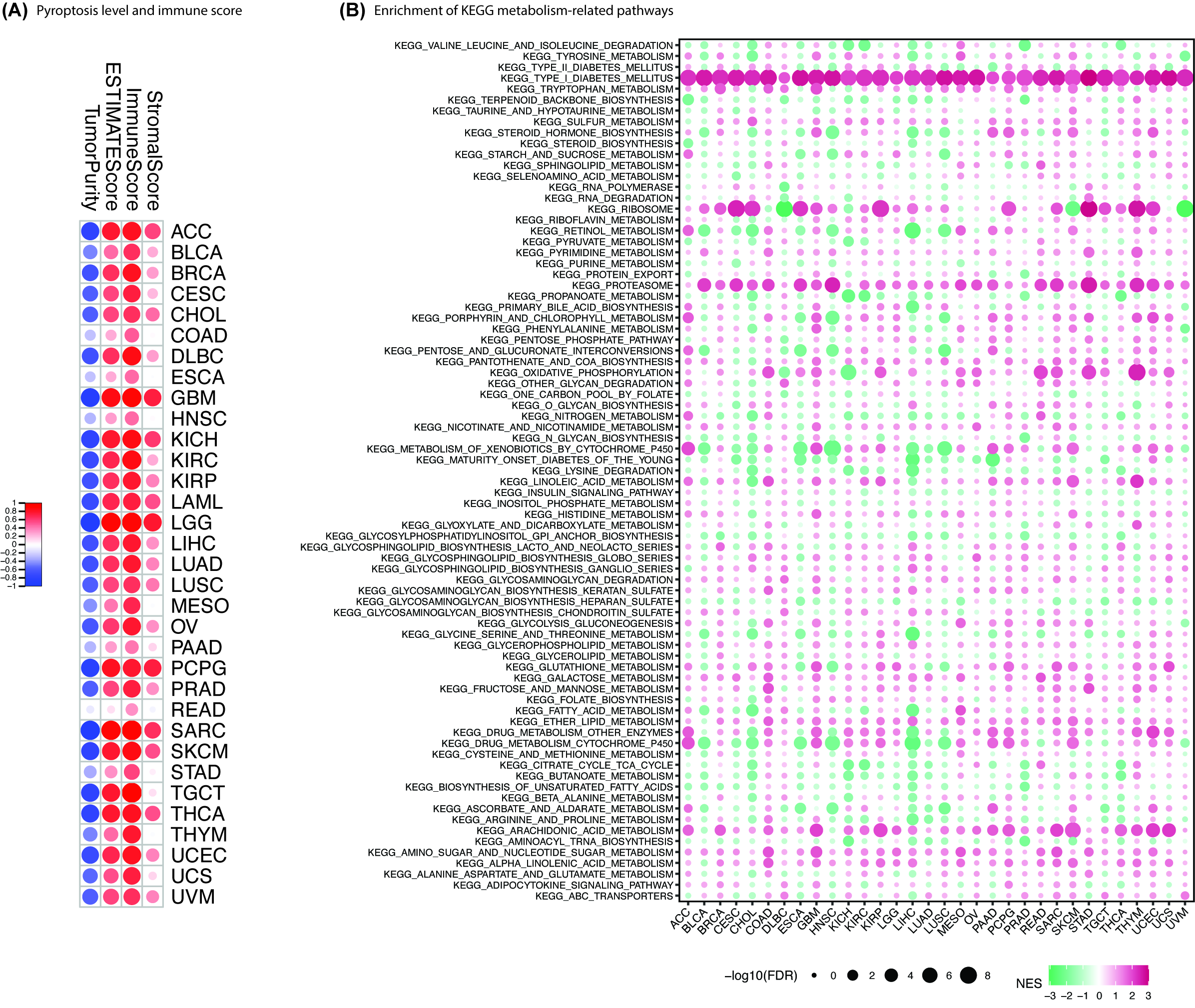


**Supplementary Figure 3. Immune features and metabolic pathways enrichment analysis.**

(A) Heatmap shows spearman correlation between pyroptosis level and ESTIMATE features. Red & blue indicate positive & negative correlation.

(B) Enrichment analysis for KEGG metabolic pathways between pyroptosis-high and pyroptosis-low tumor tissues. NES is the normalized enrichment score in the GSEA algorithm.
